# Advancing sustainable rice production in the Vietnamese Mekong Delta insights from ecological farming systems in An Giang Province

**DOI:** 10.1016/j.heliyon.2024.e37142

**Published:** 2024-08-29

**Authors:** Dung Duc Tran, Edward Park, Can Thu Van, Thien Duc Nguyen, Au Hai Nguyen, Tran Che Linh, Pham Hong Quyen, Duong Anh Tran, Hong Quan Nguyen

**Affiliations:** a National Institute of Education, Earth Observatory of Singapore, and Asian School of the Environment, Nanyang Technological University, Singapore; b Center of Water Management and Climate Change, Institute for Environment and Resources, Vietnam National University – Ho Chi Minh City (VNU – HCM), Ho Chi Minh City, Viet Nam; c University of Natural Resources and Environment, Vietnam National University – Ho Chi Minh City (VNU – HCM), Ho Chi Minh City, Viet Nam; d Institute for Environment and Resources, Vietnam National University – Ho Chi Minh City (VNU – HCM), Ho Chi Minh City, Viet Nam; e Department of Agriculture and Rural Development, An Giang Province, Viet Nam; f Laboratory of Environmental Sciences and Climate Change, Institute for Computational Science and Artificial Intelligence, Van Lang University, Ho Chi Minh City, Viet Nam; g Faculty of Environment, School of Technology, Van Lang University, Ho Chi Minh City, Viet Nam; h Institute for Circular Economy Development, Vietnam National University – Ho Chi Minh City (VNU – HCM), Ho Chi Minh City, Viet Nam

**Keywords:** Ecosystem, Sustainable, Floodplain, Vulnerability, Water quality

## Abstract

Rice serves as a crucial staple food crop for half of the world's population. In the Vietnamese Mekong Delta (VMD), rice production plays a vital role in national food security. However, the majority of the existing intensified rice cultivation schemes in the VMD, which are typically traditional, have rendered many farmers' livelihoods unsustainable due to issues such as land degradation, water pollution, health risks, and low profitability. Therefore, it is imperative to explore alternative sustainable farming systems. This study investigates the benefits of two ecological farming systems, specifically organic rice and rice mixed with lotus, as alternatives to conventional rice farming in the upper VMD floodplain province of An Giang. These two farming systems have demonstrated long-term socioecological and economic advantages. On the one hand, they allow the introduction of rice products to the market at more affordable prices. Additionally, they contribute to improved water quality, improved soil fertility, and increased biodiversity such as bird, fish, and plant species compared to traditional rice farming systems. Although we acknowledge that the availability of floodwater poses a significant constraint for alternative farming systems, the business opportunities and socioecological benefits associated with these systems outweigh the limitations. Our findings provide evidence that ecological farming practices that support rice cultivation represent promising alternatives to sustainable rice production, which can help mitigate vulnerabilities in intensified rice farming systems and can be scaled up for other floodplain provinces in the VMD and beyond.

## 1 Introduction

Rice is the main food for half of the world's population, and its importance is recognized in hunger reduction, particularly in Asia [1]. Despite the benefits of rice in food security, the extensive development of intensive rice farming systems has degraded soil conditions, polluted water, and caused environmental risks in many regions, particularly the developing countries [[2], [3], [4], [5]] due to the overuse of agrochemicals for increasing yields [[6], [7], [8], [9]]. Many studies have considered the above-mentioned problems to investigate alternative farming systems, which not only increase products and profits without being harmful to the environment but also provide long-term benefits to farmers [4,[10], [11], [12]].

Rice production in the Vietnamese Mekong Delta (VMD) not only ensures the livelihoods of about 18 million residents of the delta, but also plays a crucial role in Vietnam's food security [13]. The region covers an area of 3.9 million hectares, equivalent to 5 % of the total space of the river basin, but it contributes 70 % of rice exports from 51 % of the national rice production, making Vietnam one of the world's most important rice exporters [14,15]. Despite such a promising production, the delta faces multiple threats related to water. On the one hand, it has experienced abnormal changes in annual floods that significantly decrease floodwater flows to maintain floodplains (so-called Long Xuyen Quadrangle and Plain of Reeds), which used to cover more than 50 % of the total delta area in historical wet seasons [16]. On the other hand, floods recently have become unpredictable, but may increase in the future due to three main reasons: (i) the uncertainties associated with upstream human activities such as hydropower dam construction and irrigation [17,18]; (ii) precipitation and rainfall pattern fluctuation due to climate change [19,20]; and (iii) the consequence of building a dike system for intensive agricultural (rice) development [13,21,22]. Of these three, intensive agricultural (rice) production has been extensively developed in high-dike protection areas, which could be managed by alternatives compared to two others but has caused land degradation and water pollution that undermine local agricultural-based ecosystem services [23,24].

Due to the intensification of rice induced by national food security policy, the floodplains in the upper parts of the VMD have changed in agroecology from a seasonal floodplain to a triple rice farming system protected by large-scale flood-control structures [25,26]. The development of dikes for triple rice protection has not only decreased yield of the first two rice crops and interrupted fertile sedimentation provided by floodwater entering the fields, but also increased costs of fertilizer use and flood protection measures such as dike and sluice gates. Furthermore, soil degradation and water pollution increase significantly in the upper floodplains, where they are considered the most important region in the VMD in terms of agriculture and economy [27,28]. For example, Howie [29] and Dung Duc Tran et al. [25] detected a decline of rice yield and an increase in fertilizer use inside the high dike area compared to the low dike areas in An Giang Province. In another study, Patro et al. [30] revealed that flood protection structures such as dikes and sluice gates only reduce the flooding problem to a certain extent while cause more complications in environmental and agro-economic aspects like water pollution and land degradation due to interrupted interconnection between fields and river systems. Similarly, Temmerman et al. [31] showed that structural measures are not well adapted to climate change impacts and are costly in terms of maintenance and operation, while restoration measures under ecosystem-based flood protection could bring long-term sustainability. Recently, Duc Tran et al. [32] concluded that the cost of triple rice production is higher than usual estimates due to the additional payments from the health risk from rice consumption and farming activities.

Research on exploring effective adaptation measures in agricultural development is needed to improve flood storage upstream and reduce land degradation due to triple rice production under dike protection for the VMD [14]. The need for dike constructions has been assessed in economic terms to identify the most appropriate adaptation measures in agricultural systems based on agriculture. Huu [33] focused on field surveys to find that the dike system has become a great threat to sustainable development in terms of water pollution, natural fish exhaustion, reduction in soil fertility, erosion, and in some cases, also due to increasing field flooding. Furthermore, Trieu et al. [34] conducted a research on “living with floods” to identify adaptation strategies and concluded that nonstructural adaptation measures are mainly approved by farmers in the An Giang and Dong Thap Provinces. Thao [35] stated that expanding the lake is the best adaptation measure to give room for river using multi-criteria analyses. Kousky and Walls [36] investigated cost-benefit analyses to conclude that land use changes and floodplain conservation based on water-related structures are vulnerable to climate change and sea level rise in the long run. Moreover, Dung et al. [3] proposed alternatives in farming systems such as rice-lotus and rice-fish for double and triple rice practice that are potentially adapted well to dike systems in the VMD floodplains. Recently, Vo et al. [37] emphasized the importance of the rice-lotus farming model as a promising alternative in the low dike areas of the VMD. They highlighted how lotus plants thrive under extremely poor natural conditions, making them well-suited for changing environments under climate variability. Additionally, lotus actively contributes to soil purification and reclamation, thereby enhancing agricultural potential in these areas. These studies however focused on large-scale alternatives and shown their limitations in investigating specific alternative farming systems better than rice associated with detailed sustainable evaluations of soil and water quality to determine agriculture adaptation strategies for the delta in the long-term development.

Our study aims to evaluate alternative agriculture systems in the floodplains of the VMD that offer superior advantages compared to existing traditional rice production systems, which are protected by the dike infrastructure. These alternative systems are expected to enhance the benefits of farmers and mitigate environmental risks associated with the excessive use of pesticides and fertilizers, thereby reducing health risks for farmers. To achieve this objective, we investigated two ecological farming systems, namely organic rice and lotus mixed with rice, as potential replacements for the current traditional intensive rice farms. We conducted a survey of the quality of water and soil in the areas where ecological rice farming was practiced in An Giang province, in order to analyze the economic and environmental benefits compared to conventional rice farming methods. Additionally, we evaluated the socioeconomic and environmental aspects of mixed lotus rice farms developed in Dong Thap Province. The findings of our study provide valuable evidence to support the implementation of adaptation strategies for the development of ecological and nature-based farming systems throughout the VMD in the future.

## 2 Methodology

### 2.1 Investigations of two ecological farms

Our study examined two crop models as alternatives to traditional rice farming practices in the delta floodplains, considering their feasibility of application and potential for expansion. First, an ecological rice farming system was owned by a highly successful male farmer in An Phu District of An Giang Province who had more than 30 years of experience in rice cultivation. Second, we evaluated the benefits of a mixed farming system that involved lotus cultivation along with rice production. To evaluate the environmental benefits for the ecological rice farm, we collected soil and water samples to analyze improvements in conditions compared to the traditional rice crop. For the lotus rice crop, we conducted a comprehensive cost and benefit analysis, considering both direct and indirect products derived from this system.

*Ecological rice farm*: We refer to this farming system as an ecological rice farm because it incorporates various practices such as integrated pest management, reduced fertilizer usage in favor of organic alternatives, absence of pesticides, implementation of advanced seed and planting technologies, water conservation methods, and promotion of natural enemies (e.g., spiders, ants) for pest control. To evaluate its effectiveness, we conducted soil and water sampling analyses, aiming to compare the impact of agrochemical usage with traditional rice farming methods. Our investigation builds upon the findings of the previous study by Tran et al. [13], which highlighted a trend of increased agrochemical usage over the years, leading to land degradation. This conclusion was drawn from data gathered through 127 interviews with double and triple rice farmers conducted in 2014 and 2016.

**Fig. 1:**
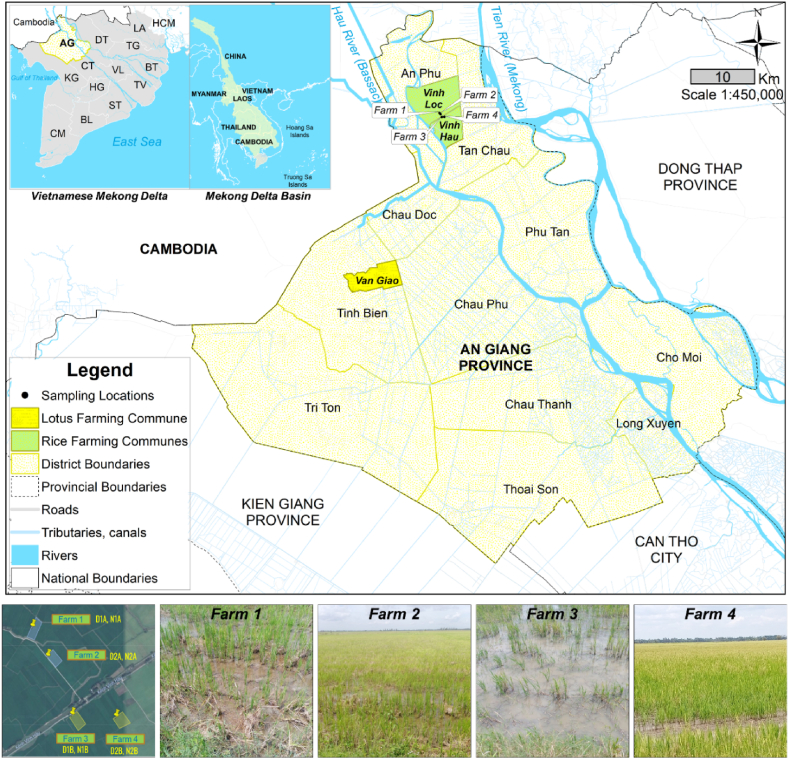
Sampling locations and farm sites in the An Giang Province of the VMD.

*Lotus mixed with rice production*: In this study, we examined a lotus rice farming system in An Giang Province developed by Eco Eco Institute (EEI) collaborated with the International Union for Conservation of Nature in Vietnam (IUCN). IUCN and EEI initially organized a training course on drawing lotus silk to teach the profession of drawing lotus silk at the request of local authorities, one of them in Van Giao commune of Tinh Bien District, An Giang Province (Fig. 1), in 2019. The livelihood model of growing lotus during the flood season (July to November annually) presents a transformative shift from the traditional rice practice of cultivating 2–3 rice crops annually. It entails cultivating a single winter-spring rice crop (December to March) while simultaneously incorporating lotus cultivation and natural fish farming during the subsequent crop cycles, namely the summer-autumn crop (April to July) and flood season. Additionally, initial steps have been taken to develop tourism during the floating season. This integrated model not only improves farmers' income, but also plays a crucial role in flood season livelihood strategies.

### 2.2 Data collection and analysis method

Our study uses a descriptive analysis method for both ecological rice farm (soil and water samplings) and lotus rice farm for qualitative assessment.

#### 2.2.1 Ecological rice farm

To evaluate the environmental benefits of ecological rice farming compared to traditional methods, we conducted soil and water samplings in one day on August 28, 2020 from four (04) rice farms at Vinh Loc and Vinh Hau communes of An Phu District, as illustrated in Fig. 1. Therefore, eight sampling points were investigated. Of those, four soil samples were obtained from farms practicing ecological rice cultivation (D1A and D2A) and four from farms employing traditional rice production (D1B and D2B). Furthermore, we collected four water samples, namely N1A and N2A from farms 1 and 2, and N1B and N2B from farms 3 and 4. This comprehensive approach allowed us to make a clear comparison between the ecological rice fields that did not use pesticides (D1A, D2A, N1A, N2A) and those where pesticides were used (D1B, D2B, N1B, N2B).

*Sampling method*: In farms 1 (D1A, N1A) and 2 (D2A, N2A), each plot is approximately 1 ha wide, the field surface is dry, and rice was harvested for four weeks. In farms 3 ((D1B, N1B) and 4 (D2B, N2B), rice was harvested for six weeks, randomly sampled and combined at a depth of 30 cm. For each soil sample, we randomized and merged soil pieces from 15 soil holes dug at random locations to combine into the same sample. For the water samples, surface water was taken at an average of 1 L and frozen for laboratory processing/experiment.

*Experimental method*: The experimental setup was carried out by Centre of Agricultural Analysis and Services in Ho Chi Minh City. We performed an analysis of various critical components, including organic matter, total nitrogen, pH, phosphorus (P), nitrogen (N), manganese (Mn), copper (Cu), zinc (Zn), and magnesium (Mg). The components were selected for assessing cultivation soil and water quality based on the standard of Vietnam under Ministry of Natural Resources and Environment (Circular 60–2015/BTNMT, QCVN No.03-2008/BTNMT, and QCVN No.08-MT:2015/BTNMT) and international references [38,39]. For organic matter, total nitrogen, pH, phosphorus (P), nitrogen (N), experimental procedures based on Vietnam Standards of 8941:2011, 6498:1999, 5979:2007, 8940:2011, and 6498:1999 were respectively applied with a soil nutrient fast detector (TPY-6A model). These standards require to all types of soil samples were air-dried before the experiment. Specifically, we dried soil samples with a volume of 100 cm3 at 105 °C for 8–10 h before scale an amount for each experiment. For instance, to measure pH with the Takemura DM15 device model, approximately 0.1 kg of the sample was used and dissolved in 500 ml of water. By settling for about 30 min, we measured the pH of the obtained water sample using a pH meter and recorded the result. To analyze heavy metals following Vietnam Standard 6496:2009 (ISO 11047:1998), 1 g of the milled sample was subjected to dry ashing at 500 °C for 12 h. The residue was then extracted with 10 ml of 1:1 nitric acid (HNO3) for 3 h, followed by filtration. The concentrations of Mn, Cu, Fe, Mg, and Zn were measured using inductively coupled plasma mass spectrometry (ICP-MS). For each component, three measurements were performed to obtain an average value. If the measurements were much different, the experiments were repeated to ensure accuracy.

#### 2.2.2 Lotus mixed with rice production

In May 2019, we interviewed 25 lotus rice farmers in Tri Ton and Tinh Bien districts using a questionnaire to implement cost and benefit analysis. This questionnaire includes three key sections of (i) farmers’ characteristics, (ii) crop conditions with cost and profit, and (iii) perspective about the flood season. Based on the questionnaire, we aimed to understand how much profit the farm provided to farmers and how the local livelihoods increase their resilience and adaptation capacity under the internal and external impacts. To measure the social and environmental benefits, we examined an average of 1 ha of lotus in the farming system developed by IUCN and Eco Eco Institute in the Tri Ton and Tinh Bien districts to support 15 local women in a project. Each hectare of lotus needs four (04) direct workers (02 men plus 02 women) and four (04) indirect workers (01 men plus 03 women).

## 3 Results

### 3.1 Soil and water quality assessment of the ecological rice farm

**Fig. 2:**
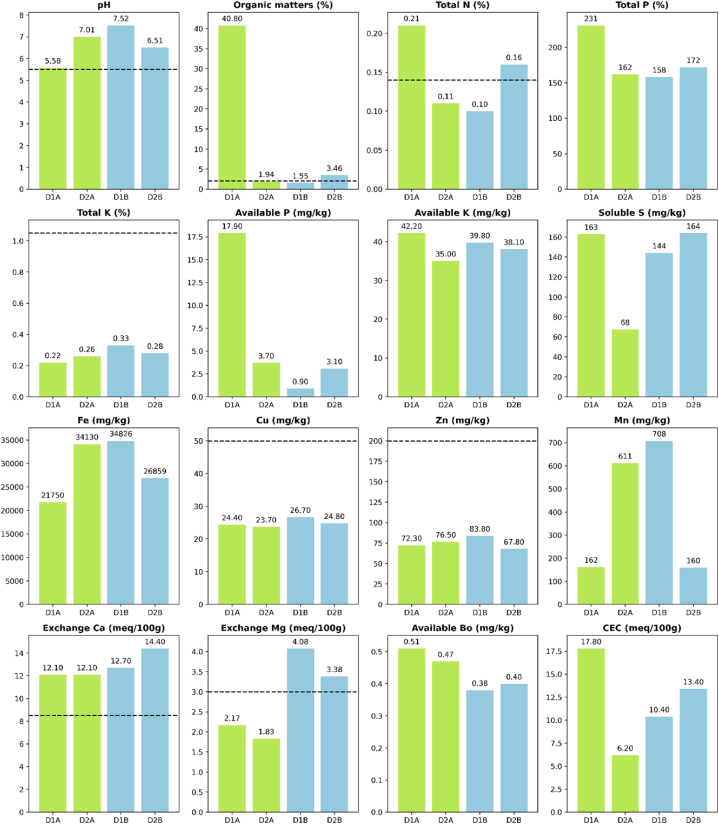
Soil sampling results of the ecological rice model (green) compared to the traditional rice model (blue). The black dot line shows national allowable standards for agricultural soils. D1A and D2A are soil samples collected from the ecological rice farms and D1B and D2B from the traditional rice farms.

**Fig. 3:**
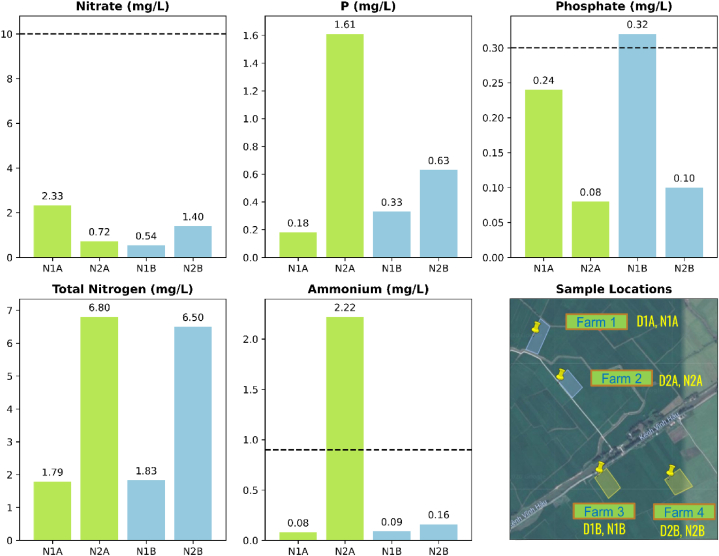
Water sampling results of the ecological rice model (green) compared with the traditional rice model (blue). The black dot line shows national allowable standards for irrigation water. N1A and N2A are water samples collected from farms 1 and 2 (ecological rice) while N1B and N2B from farms 3 and 4 (traditional rice).

The soil and water sampling results presented in Fig. 2, Fig. 3 indicate the average values of the samples collected in four (04) farms. Compared to the traditional rice farm, soil samples from ecological/organic farms have higher organic matter (8 times), total nitrogen (0.180 % compared to 0.125 %), total and available phosphorus and available Bo (mg/kg). Similarly, we found higher potassium, soluble sulfate, Fe, Cu, Mn, and exchange Mg in the samples of the traditional farms. Values are higher than national standard limits found from pH, organic matter, total nitrogen, Ca, and Mg. For water samples, ammonium, P, and total nitrogen are higher in ecological/organic farms, while Phosphate is lower. Phosphate and ammonium are higher than the national standard in samples of N1B and N2A.

The pH value of the soil falls within the neutral range, indicating favorable soil conditions for D1A soil sampling. The substantial variation in quality, with a richness 20 times higher than average, further confirms the excellent soil quality of this sample. Furthermore, upon observation, it is evident that the soil is dry and there are no visible indications of the use of organic fertilizers by the farmer who owns the land. The soil analysis reveals abundant levels of essential nutrients, including nitrogen (N), phosphorus (P) and potassium (K). However, the reliability of phosphorus availability is uncertain and elevated levels of heavy metals, such as copper (Cu) and zinc (Zn), have been detected. Despite these concerns, there is a positive aspect to highlight: the soil shows substantial exchange of the inorganic nutrient calcium (Ca), suggesting an environment conducive to efficient uptake of nutrients by plants. The soil's nutrient adsorption capacity, also known as Cation Exchange Capacity (CEC), is classified as medium. This suggests that the soil has a commendable ability to retain nutrients and minimize nutrient drift, which is beneficial for plant nutrition. However, it should be acknowledged that soil capacity to release nutrients quickly is limited, reducing the need for frequent analysis and monitoring.

For D1B soil sampling, an alkaline pH level was found to contain an average amount of organic matter. This can be attributed to the timing of soil collection, which occurred after the growing season. The soil nutrition, which encompasses nitrogen (N), phosphorus (P) and potassium (K), is moderately average. However, the reliability of P is uncertain, whereas the levels of heavy metals, such as copper (Cu) and zinc (Zn), are all below the acceptable threshold. In contrast, soil exhibits a high level of inorganic calcium (Ca) exchange, promoting efficient nutrient uptake by plants. Soil shows low nutrient adsorption capacity, which signifies limited ability to retain essential nutrients, making it prone to erosion through water runoff. As a result, the soil tends to degrade rapidly under these circumstances.

The soil is characterized by a notably high alkaline pH level, accompanied by an average organic matter content found in D2A sampling. These characteristics can be attributed to the influence of post-seasonal factors and conditions affecting soil composition. Soil nutrition, which includes nitrogen (N), phosphorus (P) and potassium (K), is highly satisfactory. However, caution should be exercised regarding the reliability of the phosphorus index (P-index). On a positive note, the levels of heavy metals, such as copper (Cu) and zinc (Zn), are all within the permissible limits. In addition, soils demonstrate a rich concentration of inorganic calcium (Ca) exchange, greatly facilitating the absorption of nutrients by plants. The soil demonstrates an average nutrient adsorption capacity (CEC), highlighting its ability to effectively retain essential nutrients and minimize washing. Consequently, the soil exhibits a relatively limited propensity for rapid depletion of nutrients, reducing the need for frequent fertilization.

For D2B sampling, the pH value of the sample falls within the neutral range, indicating a balanced acidity level. However, it should be noted that this sample exhibits a significant amount of organic matter. This observation aligns with the findings from our field survey, where we discovered that farmers often leave behind the by-products of their harvest on the field. This practice contributes to a notable increase in the organic content of the soil. The soil nutrition, which includes essential elements such as nitrogen (N), phosphorus (P), and potassium (K), has favorable levels. It should be noted that the reliability of the phosphorus (P) value is questionable. However, an encouraging aspect is that the levels of heavy metals, including copper (Cu) and zinc (Zn), remain well below the permissible threshold. Additionally, the presence of inorganic nutrients, particularly calcium (Ca) exchange, is abundant, indicating a nutrient-rich environment. Such favorable soil conditions contribute to the efficient absorption of nutrients by plants, fostering their healthy growth and development. The soil exhibits an average nutrient adsorption capacity, commonly termed cation exchange capacity (CEC). This characteristic indicates that the soil has a commendable ability to retain nutrients, minimizing the risk of leaching or washing of nutrients. Moreover, this attribute implies that the soil has a reduced tendency to rapidly deplete its nutrient content, thus requiring fewer fertilization interventions. Consequently, the inherent capacity of soils to retain nutrients contributes to its sustained fertility and reduces the need for frequent fertilizer applications.

For water sampling, most of the water samples obtained exhibit a quality suitable for irrigation purposes. However, it is worth noting that sample N2A stands out due to its elevated ammonium content, likely influenced by the presence of animal wastewater. On the other hand, sample N1B exceeds the allowed threshold for phosphate levels, indicating the use of chemical fertilizers. These findings serve as evidence of agricultural practices involving the application of chemical fertilizers and the potential impact they have on water quality.

### 3.2 Sustainability assessments

#### 3.2.1 Cost and benefit analysis (CBA) of the ecological rice farm

The biosecurity-based ecological rice production model was implemented in winter-spring 2017–2018, with the cost and benefit results shown in Table 1. We found a reduction of investment cost of 376.9 $US (630.4 $US compared to 1007.3 $US) equivalent to 7,586,000 VND/ha (1 $US equals 20,130 VND in 2017) due to an increase in yield of 0.35 tons/ha. The total net profit is 399.4 $US/ha (∼8,040,000 VND/ha), which is 39.7 % higher than the traditional rice crop.

**Table 1:** Cost and profit between ecological rice model (ERM) and traditional rice model (TRM).

Value	ERM ($US)/ha	TRM ($US)/ha	Comparison ($US)/ha
**Investment Cost**	630.4	1007.3	376.9 *(∼7,586,000 VND/ha)*
**Revenue**	2035.6	2013.1	
**Net Profit**	1405.2	1005.8	399.4 *(∼8,040,000 VND/ha)*

Exchange rate (2017): 1 $US = 20,130 VND.

#### 3.2.2 CBA and socio-environmental benefits of the lotus-rice farm

The lotus planting model in An Giang Province is applied on rice land deployed in crop rotation. Instead of farmers producing two to three rice crops per year, with this farming system, farmers only produce one winter-spring rice crop, one summer-autumn rice crop, and grow lotus for the third crop during the flooding season. According to the above profit analysis, farmers earn around 2724 $US/year (54,835,000 VND/year by 51,700,000 VND/year plus 3,135,000 VND/year) for 1 ha of land from the lotus planting model (Table 2). Furthermore, in the winter-spring rice crop, farmers add an average income of about 1242 $US/ha (25,000,000 VND/ha). If farmers plant the summer-autumn rice crop, the average profit of the summer-autumn rice crop is approximately 745 $US/ha (∼15,000,000 VND). In addition, farmers have additional income from the by-products of the lotus stem that is used to make lotus silk. Overall, the lotus rice model can provide a double profit compared to that of the only rice farm (3966 $US compared to 1987 $US).

**Table 2:** Profit comparison between the lotus-rice model and the mono-rice model.

No	Contents	Only rice (VND)	01 winter-spring rice crop + summer-autumn lotus season and floating season (VND)
*1*	Benefit from winter-spring rice	25,000,000 (∼1242 $US)	25,000,000 (∼1242 $US)
*2*	Benefit from summer-autumn rice	15,000,000 (∼745 $US)	0
*3*	Benefit from lotus	0	51,700,000 (∼2568 $US)
*4*	Benefit from fish	0	3,135,000 (∼156 $US)
*5*	Benefit from lotus silk	0	
***Total (VND)***		**40,000,000**	**79,835,000**
***($US)***		**1987 $US**	**3966 $US**

Exchange rate (2017): 1 $US = 20,130 VND.

#### 3.2.3 Social and environmental benefits

According to lotus farmers, raising fish and planting trees in the flood season are also activities familiar to people in rural areas. There are no machines to plant and harvest lotus; most of them are manual and labor-intensive; therefore, activities related to the lotus model can create many jobs for rural people, especially poor households and unemployed women. Lotus activities are also simple, easy to do, and close to their homes; particularly women and rural people can initially open up community tourism activities in their villages. This is an idea of what people and local movements think. In addition, local livelihoods help women have the opportunity to take care of their families and children well, avoiding migrating away from their homes to urban areas to earn a living. Furthermore, the lotus model has created a typical livelihood model in the flood season, providing higher income than traditionally intensive farming of two or three rice crops per year. The success of the model is a pilot to replicate to neighboring areas with similar conditions, thereby helping to encourage rice farmers to change farming patterns during the flood season, giving rural jobs during the flood season, and generating daily income from lotus.

The lotus silk weaving model helps lotus growers take advantage of the lotus stem as a by-product. Instead of discarding it, lotus silk produces a clean product, becoming a raw material for the production of very soft, light, and environmentally friendly fabrics. A lotus silk weaving is almost suitable for women in rural areas, leisurely farmers, helping women to have more incomes, more income in their leisure time, helping women to have more jobs at home, and no glass of incense. They can stay at home to work while caring for their children and their families, and earn more income, especially partly solving the unemployment of female laborers in rural areas.

The lotus-rice farm not only brings economic and social benefits, but also helps the district and provincial governments implement the policy of changing crop structure, improving soil and improving the environment and biodiversity. First, it creates new environmentally friendly products from clean rice (only 01 rice crop per year - less use of pesticides and fertilizers) and pure lotus seeds, attracting more wild fish. In addition, this farm creates a diverse ecological environment, attracting many birds, such as white storks. Second, through extensive consultation and interviews with farmers cultivating nearby lotus fields, they said that the rice-lotus farm is a suitable livelihood model in the flood season, particularly in 2018 when the flooding rose quickly and receded early. The lotus plant is ideal for following up with floodwater, while lotus fields receive an average alluvium yearly from 0.5 to 0.7 which improves soil quality for the next winter-spring rice crop.

## 4 Discussion

### 4.1 Potential to improve ecological/organic agriculture systems in delta floodplains

Floodwater regimes and floods have changed across the VMD floodplains that need serious attention to land use change to adapt to new hydrological conditions toward sustainable development [19,22,[40], [41], [42], [43]]. In general, the decrease in the flood area should be taken into account to increase flood-based farming systems in the future, while ecological rice farms are alternatives to replace on a larger scale the existing traditional triple rice which has shown its failure in cultivation methods of overuse of agrochemicals negatively affecting land and water quality [17,28,44]. Transformation progress takes time since traditional rice production is long-term cultivation and farmers are only willing to change their crops when they find the success of alternatives such as durable high yield and price with a stable market [13,21]. Clearly, the benefits of ecological rice and lotus-rice farms as revealed by our research findings indicate economic, social, and ecological aspects that could help farmers reduce their health risk and sustain livelihoods in the long terms [32]. Therefore, the sustainability of farmer livelihoods is improved with the benefits of alternative farming systems in the short term to change their transformation perspective.

Two farming systems investigated in our study, which show much higher socio-economic and environmental benefits than traditional rice practices, could be widely applied on a large scale in delta floodplains with changes in hydrological condition changes [28]. In terms of environmental benefits, ecological rice farm has much higher nutrient and valuable organic matter in soil and water based on our sampling analysis. While the ecological rice crop could feasibly replace the traditional rice crop (double and triple rice crop patterns) in existing low and high dike areas, developing a lotus farm on a large scale is more difficult. However, lotus farmers said that lotus plants still live well in rice soil and this is a fragment of concrete evidence to enhance this crop across floodplains in areas protected by the dike. At the same time, this model is grown well under water depths of 0.9–1.5 m, consistent with the province's policy of transforming crop structure in recent years with water conditions in the delta floodplains [3]. Currently, this lotus growth model is being replicated in other districts of An Giang Province, such as in the watershed area of An Phu District. This area is part of the "Sustainable Livelihood Transformation" project funded by the World Bank, and by 2025, the lotus area is expected to expand to 500 ha. The lotus-growing model can take advantage of the land area in low-lying areas that are dominant in many locations of the VMD. Overall, the two alternative farming systems of rice and lotus not only increase household economic income (double compared to only rice model) and create more jobs for rural people, but also contribute to improving the landscape and ecological environment in rural areas, promoting the development of more secondary services such as tourism or local culture, etc. These systems use much less pesticides and fertilizers that reduce production costs and gain much higher profits and minimize health risks and health costs of many farmers [45] to better improve their livelihood sustainability and improve resilience to adapt with increasing changes in climate and environmental pressures [[46], [47], [48], [49]].

### 4.2 Policy implications for adaptation measures

It is crucial to develop integrated empirical frameworks and regulations involved in agricultural water management strategies across delta scales, which underline the importance of more friendly ecological (such as ecological rice crop) and flood-based farming systems (lotus crop mixed with rice) to adaptation strategies for sustainable development of the delta [27,50]. These frameworks and regulations not only take into account national and international dynamics, but also the specific local systems, paying attention not only to temporal scale (changes over years based on the conditions of the water regime in the VMD) but also spatial level (changes due to external impacts from developments of hydroelectric dams upstream of the Mekong River and climate change). In fact, the Resolution 120 proposed in 2017 has raised regulations supporting natural-based measures for adaptation strategies adopted in agricultural production within the floodplains of the VMD and beyond [28,51]; however, we need many successful pilots to prove that natural-based farming systems have much higher benefits to farmers in the long term.

In addition to frameworks and regulations, practical pilots for adopting ecological or flood-based farming systems are necessary to succeed [34,52]. It is also important to involve different stakeholders (scientists, farmers, local officials, and companies) in the pilots to improve social impacts based on their strengths. Another promising activity to consider is the dissemination of successful stories in media systems to increase awareness and perception of farmers and related stakeholders. This has important implications for resolving the points of view of many rice farmers who want to keep their existing traditional rice farming systems and promote cooperation between the stakeholders [53,54]. Additionally, governments should provide different programs, such as low-interest loans and agricultural extension programs, to regularly support farmers to invest in new farms and learn new technologies to transform their existing farming systems into higher beneficial ones [55,56]. In this context, training programs should be enhanced to train farmers in new farming systems such as best management practice that apply much less pesticides and fertilizer [57,58]. Furthermore, enhancing the cooperation between farmers and enterprises is essential to ensure that agricultural products are of high quality to be consumed on national and international markets [59]. Furthermore, the government should advance food processing technology involving the food value chain and supply, which still needs to be better applied in the VMD and Vietnam [60,61].

In the VMD floodplains, most farmers have tried to find ways to maintain their rice-based crops, although the top-down policy has been applied to change other economic crops. Instead of the dominance of top-down policy, multiscale government should advance bottom-up policies at the same time to better understand the failure of the governmental system [33,62]. This has been challenging in reaching an optimal farming model for farmers involved in policy-to-action. That is the reason why farmers continue rice cultivation and develop triple rice practice to increase their income because only rice has been secured by the government-supported market. In addition, the long-term governmental strategy to support rice production in quantity has forced rice farmers to convince their businesses instead of conversion of land use to other crops with risk and low capacity. For example, according to local officials and farmers who directly grow lotus, the conversion from the monoculture rice model to the planting of one winter-spring rice crop in combination with lotus cultivation gives higher incomes, but the experience of farmers should be more advanced and needs support from the local government in policy making that create loans programs and develop stable markets better than rice. Therefore, effective and feasible policies and strategies play an important role in improving the success of transformation.

### 4.3 Limitations and future outlooks

While our efforts have been directed towards highlighting the benefits of two alternative farming models in the floodplains of the VMD, it is essential to acknowledge two limitations. Firstly, our investigation has primarily focused on An Giang Province, therefore further case studies in various provinces are warranted to ensure the comprehensive representation of farming practices across the entirety of the delta floodplains. This would provide a more holistic understanding of the applicability and efficacy of these models across different geographic and socio-economic contexts within the VMD. Secondly, it is crucial to note that our assessment of soil and water quality in the ecological rice farm was conducted only once. To derive robust conclusions and facilitate meaningful comparisons, it is imperative to undertake a series of measurements across different seasons. In addition, soil parameters and water quality of lotus cultivation farming system could be collected to help us compare to those of a traditional rice crop over the three-season cultivation. This longitudinal approach will enable us to capture variations in environmental parameters over time, allowing for a more nuanced analysis and the formulation of concrete findings essential for drawing informed conclusions.

## 5 Conclusions

This study aims to explore ecologically friendly and sustainable flood-based agriculture systems that can offer a wide range of benefits to communities residing in the Vietnam Mekong Delta (VMD). Two alternative models studied are expected to promote more sustainable livelihoods for local farmers and improve their resilience and adaptive capacity in the face of both internal factors such as land degradation and water pollution resulting from the dike infrastructure that supports intensive rice production, as well as external impacts arising from the construction of hydroelectric dams upstream of the Mekong River. Based on the findings, we further investigate the potential large-scale applications of these farming systems to improve flood retention capacity and foster diverse ecosystems within floodplains. The study draws two main conclusions:

• Farmers residing in the floodplains of the VMD have been working to diversify their farming systems in response to hydrological changes, such as decreased flood levels in recent years, which are attributed to the impacts of climate change and the development of hydropower dams upstream of the Mekong River. Despite these challenges, many farmers have remained faithful to traditional rice practices, namely double and triple rice cultivation, which are considered less ecologically friendly. This decision is primarily driven by the stable local market for rice and the limited capacity of farmers in terms of economic investment and technology. However, farmers have gradually become aware of the negative environmental impacts associated with these practices over time. The implementation of more environmentally friendly approaches, such as ecological rice and flood-based agriculture systems that incorporate lotus mixed with rice, provides compelling evidence that floodplains have the potential to explore various farming options. These alternatives not only offer opportunities for increased income but also yield long-term environmental and social benefits. By adopting these systems, farmers can reduce health risks and generate additional employment opportunities. In comparison, intensive rice production, particularly triple rice cultivation, lacks these advantages.
• The flooded areas of the VMD floodplain have been significantly affected by a range of environmental pressures, including the construction of hydroelectric dams upstream, impacts of climate change, rising sea levels, and extensive development of dikes in the upper floodplains of the delta and surrounding regions. Our study reveals that these environmental pressures, along with intensive rice farming practices, pose a significant challenge to the ecological conservation of the VMD floodplains. Consequently, this issue has garnered considerable attention from scientists seeking alternative solutions. It is crucial to address the farming practices of many farmers, either by cultivating crops that can effectively use floodwaters as a shared resource or by allowing flood seasons for soil rejuvenation without crop cultivation. More research is needed to explore this problem comprehensively and develop appropriate adaptive solutions for the future.

## Disclosure statement

All authors stated that there was no conflict in this article.

## Data availability

Data will be made available on reasonable request.

## CRediT authorship contribution statement

**Dung Duc Tran:** Writing – review & editing, Writing – original draft, Methodology, Data curation, Conceptualization. **Edward Park:** Writing – review & editing, Visualization, Project administration, Investigation. **Can Thu Van:** Writing – review & editing, Methodology. **Thien Duc Nguyen:** Writing – original draft, Visualization, Methodology. **Au Hai Nguyen:** Writing – review & editing, Writing – original draft, Methodology. **Tran Che Linh:** Writing – review & editing, Data curation. **Pham Hong Quyen:** Writing – review & editing, Data curation, Conceptualization. **Duong Anh Tran:** Writing – review & editing. **Hong Quan Nguyen:** Writing – original draft, Supervision, Project administration, Methodology, Conceptualization.

## Declaration of competing interest

The authors declare that they have no known competing financial interests or personal relationships that could have appeared to influence the work reported in this paper.
